# Asymmetric Cell Division of Fibroblasts is An Early Deterministic Step to Generate Elite Cells during Cell Reprogramming

**DOI:** 10.1002/advs.202003516

**Published:** 2021-02-25

**Authors:** Yang Song, Jennifer Soto, Pingping Wang, Qin An, Xuexiang Zhang, SoonGweon Hong, Luke P. Lee, Guoping Fan, Li Yang, Song Li

**Affiliations:** ^1^ Department of Bioengineering University of California Los Angeles Los Angeles CA 90095 USA; ^2^ Department of Human Genetics University of California Los Angeles Los Angeles CA 90095 USA; ^3^ Division of Engineering in Medicine Department of Medicine Brigham and Women's Hospital Harvard Medical School Boston MA 02115 USA; ^4^ Department of Bioengineering Department of Electrical Engineering and Computer Science University of California at Berkeley Berkeley CA USA; ^5^ Institute of Quantum Biophysics Department of Biophysics Sungkyunkwan University Suwon 16419 Korea; ^6^ College of Bioengineering Chongqing University Chongqing 400044 China; ^7^ Department of Medicine University of California Los Angeles Los Angeles CA 90095 USA

**Keywords:** asymmetric cell division, cell fate determination, direct reprogramming, epigenetic state

## Abstract

Cell reprogramming is considered a stochastic process, and it is not clear which cells are prone to be reprogrammed and whether a deterministic step exists. Here, asymmetric cell division (ACD) at the early stage of induced neuronal (iN) reprogramming is shown to play a deterministic role in generating elite cells for reprogramming. Within one day, fibroblasts underwent ACD, with one daughter cell being converted into an iN precursor and the other one remaining as a fibroblast. Inhibition of ACD significantly inhibited iN conversion. Moreover, the daughter cells showed asymmetric DNA segregation and histone marks during cytokinesis, and the cells inheriting newly replicated DNA strands during ACD became iN precursors. These results unravel a deterministic step at the early phase of cell reprogramming and demonstrate a novel role of ACD in cell phenotype change. This work also supports a novel hypothesis that daughter cells with newly replicated DNA strands are elite cells for reprogramming, which remains to be tested in various reprogramming processes.

## Graphical abstract

The asymmetric cell division (ACD) of fibroblasts at the early stage of reprogramming plays a key role in cell fate determination and enables the generation of “elite cells” during cell reprogramming. An “asymmetric reprogramming hypothesis” is proposed, whereby cells with newly replicated DNA strands after ACD may have more epigenetic plasticity for reprogramming.

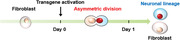

## 1 Introduction

Cell reprogramming is a powerful tool to engineer cell fate, which enables cell type conversion beyond stem cell differentiation, and has wide applications in regenerative medicine and therapeutic development.^[^ ^1^, ^2^, ^3^, ^4^ ^]^ Somatic cells can be reprogrammed into induced pluripotent stem cells (iPSCs) or directly converted into distantly related cell types, such as neurons and cardiomyocytes, by using exogenous transcription factors, small molecule compounds, and biophysical cues.^[^ ^5^, ^6^, ^7^, ^8^, ^9^, ^10^, ^11^ ^]^ Cell reprogramming is thought to consist of multi‐step epigenetic changes that involve erasing the memory of the original cell type and activating the genes of target cell types over a time period of days to weeks. However, the limiting factor for cell reprogramming is not well understood. Several models have been used to explain the low efficiency of cell reprogramming, including the “elite” cell hypothesis, stochastic process, and deterministic model,^[^ ^12^, ^13^, ^14^, ^15^ ^]^ but it is not clear how elite cells are generated and whether deterministic steps exist.

Asymmetric cell division (ACD) is an important mechanism in stem cell differentiation and cancer cell phenotype change.^[^ ^16^, ^17^, ^18^, ^19^ ^]^ It is a highly‐controlled process that regulates cell fate decisions in various cell types, including neural stem/progenitor cells, muscle satellite stem cells, hematopoietic stem cells, mammary stem cells, T‐lymphocytes, and basal epidermal cells.^[^ ^20^, ^21^, ^22^, ^23^ ^]^ During ACD, cell fate determinants (e.g., proteins, RNA, and histones) are segregated unequally into two daughter cells, enabling the change of cell fate.^[^ ^24^ ^]^ It has also been shown that, during stem cell differentiation, “immortal DNA strands” and epigenetic memory are inherited into one of two daughter cells to maintain genome stability and stemness.^[^ ^16^, ^25^ ^]^ Although the exact mechanism of ACD regulation is not fully understood, several signaling pathways, such as atypical protein kinase C (PKC) and Notch, have been shown to be involved in this process.^[^ ^26^, ^27^, ^28^, ^29^, ^30^ ^]^ However, whether ACD occurs in cell reprogramming remains unknown.

Since cell division involves DNA replication and extensive chromatin remodeling, we hypothesized that cell division could result in a quantum‐step change in chromatin and thus cell fate determination during the reprogramming process. To test this possibility, we performed a detailed analysis of early cell division following transgene expression, and unraveled an unexpected role of ACD during the direct conversion of fibroblasts into induced neuronal (iN) cells.

## 2 Results

### 2.1 Fibroblasts Undergo ACD during iN Reprogramming

**Figure 1:**
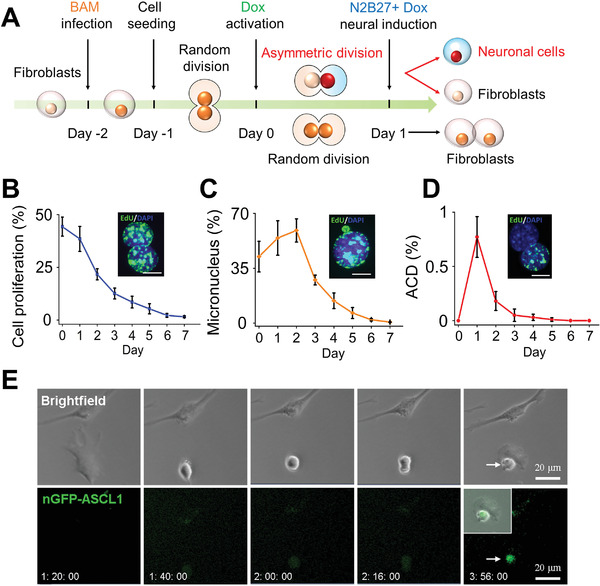
ACD of fibroblasts during iN reprogramming. A) Experimental timeline for iN conversion. B) Quantification of cell proliferation during the first week of reprogramming based on 3h EdU labeling. Representative images show proliferating cells (EdU^+^, green) and nuclei (DAPI, blue). Scale bar: 10 µm. Line graph shows mean ± SD (*n* = 4). C) The percentage of cells with micronucleus formation during the course of reprogramming. Representative image of a micronucleus adjacent to a nucleus. Scale bar: 10 µm. Line graph shows mean ± SD (*n* = 4). D) Quantification of ACD percentage during the course of reprogramming. Representative image of asymmetrically dividing daughter cells where one cell is EdU^+^ and the other is EdU^−^. Scale bar: 10 µm. Line graph shows mean ± SD (*n* = 4). E) Representative images from live‐cell time‐lapse imaging showing fibroblasts transduced with eGFP‐Ascl1, Brn2, and Myt1l undergoing ACD within 24 h after the addition of Dox. Scale bar: 20 µm.

Adult mouse fibroblasts were synchronized in the G0/G1 phase of the cell cycle, transduced with doxycycline (Dox)‐inducible lentiviral vectors containing the three reprogramming factors Brn2, Ascl1, and Myt1l (BAM),^[^ ^31^ ^]^ and seeded onto fibronectin‐coated glass coverslips the next day (**Figure** **1**A). One day later, Dox was added (marked as day 0) to induce BAM expression, and cells were cultured in serum‐free N2B27 medium for the remainder of the experiment (Figure 1A). We first examined the time course of cell proliferation during iN conversion by pulsing cells for 3 h with 5‐Ethynyl‐2’‐deoxyuridine (EdU), a thymidine analog that is incorporated into the DNA of dividing cells. The experiments revealed that cell proliferation rate was the highest during day 0 to day 1 (≈50%), and then gradually declined, which could be attributed to the absence of serum in the neuron‐induction medium (Figure 1B). At the same time, we observed extensive micronucleus formation (40–50%) within the first two days (Figure 1C), which at least in part accounted for cell death and lower reprogramming efficiency during the reprogramming process. Micronucleus formation was likely due to the disorganization and missegregation of chromosomes caused by lentiviral transduction because the lentiviral transduction of green fluorescent protein (GFP) gene induced a similar effect. Interestingly, we found that approximately 1% of the fibroblasts were dividing asymmetrically, where one daughter cell was EdU^+^ while the other was EdU^−^ (Figure 1D). On the other hand, live‐cell time‐lapse imaging of fibroblasts transduced with an nGFP‐Ascl1 fusion construct, Brn2 and Myt1l showed that, within the first day of inducing BAM by adding Dox, fibroblast division might occur with only one daughter cell expressing nGFP‐Ascl1 (Figure 1E). These findings motivated us to investigate whether ACD could cause significant changes in daughter cells that had an impact on the reprogramming process.

**Figure 2:**
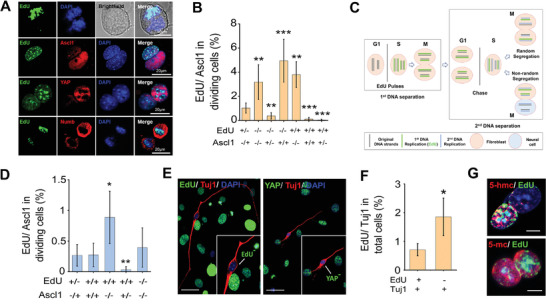
iN cells inherit newly replicated DNA strands during ACD. A) Immunofluorescent and bright‐field images of asymmetrically dividing fibroblasts stained with EdU, Ascl1, YAP, and Numb after Dox treatment for 24 h. The top panel shows asymmetrically dividing daughter cells at the mitosis stage after initiating iN reprogramming with Dox. Scale bar: 10 µm (top) or 20 µm. B) Quantitative analysis of the presence (+) or absence (−) of EdU‐labeling (based on 3‐h labeling) and Ascl1‐expression in two mitotic cells on day 1. Bar graph shows mean ± SD (*n* = 6), ** *p* ≤ 0.01, ***p ≤ 0.001. Significance was determined by a one‐way ANOVA and Tukey's multiple comparison test. C) Pulse‐chase assay to study sister chromatid segregation during cell division. All DNA strands are labeled through the administration of nucleotide analogs (e.g., EdU) over multiple generations when the cells or their precursors are dividing symmetrically. If immortal template strands exist, they would become labeled. Following the label‐free chase period, cells would retain the labeled strands even after multiple cell divisions. However, in an asymmetrically dividing cell, newly synthesized DNA strands are not labeled with EdU during the S phase of the first cell cycle. Inheritance of all the label by only one of the daughter cells in the second cell cycle would indicate that these cells inherited the parental strands. D) Fibroblasts were labeled with EdU for 3 days prior to BAM transduction. One day after initiating iN reprogramming, cells were fixed and stained for EdU and Ascl1. Quantification of the presence (+) or absence (−) of EdU‐labeling and Ascl1 expression in mitotic cells on day 1. Bar graph shows mean ± SD (*n* = 6), *p ≤ 0.05, **p ≤ 0.01. Significance was determined by a one‐way ANOVA and Tukey's multiple comparison test. E) Pulse‐chasing with EdU was performed as in (B). On day 3, cells were fixed and stained for EdU, Tuj1, and YAP. Scale bar: 50 µm. F) Quantification of the presence (+) or absence (−) of EdU‐labeling in BAM‐transduced fibroblasts expressing Tuj1 on day 3. Bar graph shows mean ± SD (*n* = 6), *p ≤ 0.05. Significance was determined by a two‐tailed, unpaired *t*‐test. G) Representative images of asymmetrically dividing fibroblasts stained for 5‐mC, 5‐hmC, and EdU on day 1. Scale bar: 10 µm.

Further analysis of early cell division at 24 h after adding Dox showed that, during ACD, EdU^+^ cells were positive for the ACD localization marker, Numb, and cell proliferation‐related marker, Yes‐associated protein (YAP), but negative for Ascl1 expression (**Figure** **2**A). In contrast, EdU^−^ cells expressed Ascl1 but not Numb or YAP, suggesting that the EdU^−^ daughter cell was primed for iN conversion (Figure 2A). Quantification of EdU‐labeling and Ascl1‐expression among dividing cells showed that Ascl1 expression was inversely correlated with EdU labeling such that almost all cells expressing Ascl1 were EdU^−^ (Figure 2B). When Ascl1 had asymmetric distribution in daughter cells, there was rarely symmetric Edu distribution, that is, Edu (−/−) or Edu (+/+). Additionally, we confirmed that the fibroblasts transduced with BAM were negative for markers of stem cells or progenitors during iN reprogramming (Figure S1, Supporting Information). It is worth noting that symmetric division could also result in two Edu^−^Ascl1^+^ daughter cells, although these cells might be different from those resulted from ACD. Therefore, we sought to determine the relative contribution of ACD to iN reprogramming.

### 2.2 The Daughter Cells Inheriting Newly Synthesized DNA Strands during ACD are Primed for iN Reprogramming

Previous studies have shown that stem cells undergo ACD resulting in daughter cells with different cell fates and distinct chromosomal DNA strands, with one daughter cell maintaining stemness by exclusively inheriting the “immortal strands” or parental chromosomes.^[^ ^16^, ^32^, ^33^ ^]^ To determine whether ACD of fibroblasts during iN conversion induced asymmetric segregation of the old or new DNA strands, we pulsed fibroblasts with EdU for 72 h to ensure all the cells were labeled with EdU before BAM induction by Dox to initialize iN reprogramming, and analyzed the expression of Ascl1 and Tuj1 on days 1 and 3, respectively (Figure 2C–F). We found a reverse correlation between Ascl1 expression and EdU labeling, where asymmetric EdU labeling (EdU+/−) corresponded to asymmetric Ascl1 expression (i.e., EdU^−^ cells expressed Ascl1) (Figure 2D), suggesting that iN precursors did not inherit the parental chromosomes (labeled by EdU). On the other hand, we also observed that symmetric division resulted in two EdU^+^/Ascl1^+^ cells (Figure 2D), suggesting both daughter cells inherited a part of parental DNA strands. On day 3, the pulse‐chase analysis further demonstrated that a significant number of Tuj1^+^ iN cells were EdU^−^ and YAP^−^, indicating that iN cells were derived from the daughter cells that did not inherit the parental DNA strands during ACD (Figure 2E,F).

In addition, high levels of DNA methylation marker 5‐hydroxymethylcytosine (5‐hmC), but not 5‐methylcytosine (5‐mC), have been shown to identify the parental strand in asymmetrically dividing stem cells.^[^ ^33^ ^]^ Immunofluorescence analysis of DNA methylation showed that there was a higher expression of 5‐hmc, but not 5‐mc, in EdU^+^ cells (stained as in Figure 2D), further demonstrating that the parental strands were inherited by the daughter cell with a fibroblast phenotype (Figure 2G).

### 2.3 Single Cell Analysis of Reprogrammed Fibroblasts in the Early Phase

**Figure 3:**
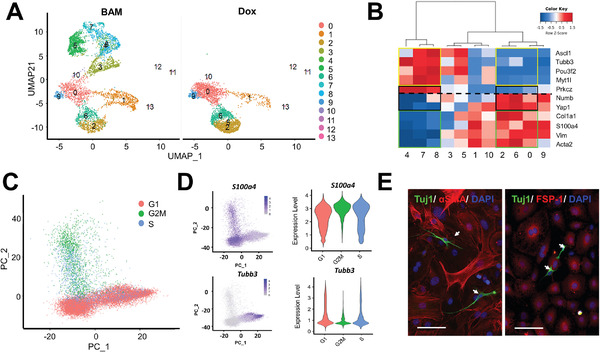
Single‐cell analysis of reprogrammed fibroblasts in the early phase. After treating non‐transduced and BAM‐transduced fibroblasts with Dox for 36 h, cells were collected for 10X single‐cell RNA sequencing analysis. A) UMAP plot analysis showing 13 cell clusters based on gene expression profile (each point represents a single cell). B) Hierarchal relationship of clusters (from A) based on the expression of neuronal, fibroblast, and ACD‐related genes. C,D) Representative PCA or violin plots of cells in different phases of the cell cycle, positive for a fibroblast marker S100a4, and positive for a neuronal marker Tubb3, respectively. E) Representative images of BAM‐transduced fibroblasts stained for *α*‐smooth muscle actin (*α*‐SMA) and fibroblast‐specific protein 1 (FSP‐1) on day 3. Tuj1^+^ iN cells are indicated by white arrows. Scale bar: 100 µm.

Since ACD mostly happened within the first two days of iN reprogramming, to characterize the heterogeneous responses of the cells at this early stage, we performed single‐cell RNA sequencing analysis of BAM‐transduced and non‐transduced fibroblasts at 36 h after Dox induction of transgenes. Uniform Manifold Approximation and Projection (UMAP) plots demonstrated the heterogeneity of the fibroblast population in the Dox‐treated sample (without BAM transduction, as control) and the new clusters (3–5, 7–8) resulted from the reprogramming process in BAM‐transduced fibroblasts (**Figure** **3**A). The hierarchal clustering clearly showed a distinct gene expression profile in these clusters (Figure S2A, Supporting Information). Since the fibroblasts were still in the early stage of reprogramming process at 36 h, single‐cell Mouse Cell Atlas (scMCA) analysis^[^ ^34^ ^]^ characterized these clusters as stromal or mesenchymal cells based on the major gene expression profiles (Figure S2B, Supporting Information). Nevertheless, the new clusters 4, 7, and 8 already showed a drastic reduction in the expression of stromal cell genes (e.g., rows 5–13). Therefore, we used a combination of neuronal genes and fibroblasts genes to characterize the clusters (Figure 3B, Figure S3, Supporting Information), which included fibroblasts (i.e., clusters 0, 1, 2, 6, 9, and 10), neuron‐like cells that partially lost fibroblast characteristics (i.e., cluster 4, 7, and 8), and the cells at intermediate stages with either low expression of fibroblast genes (i.e., cluster 3) or with the expression of both fibroblast and neuronal markers (i.e., cluster 5). Single‐cell trajectory analysis using Monocle2 confirmed these relationships among the clusters and the potential path of fibroblast conversion into neurons (Figure S4, Supporting Information).

In addition, as exemplified in Figure 3C and Figure S5, Supporting Information, principal component analysis (PCA) of single‐cell transcriptomics showed that a large proportion of cells were in the G0/G1 phase (≈70%) phase, consistent with the microscopic observation that ≈30% cells were proliferating at day 1.5 (Figure 1B). Further analysis revealed that cells expressing neuronal markers were predominantly in the G0/G1 phase, whereas cells expressing fibroblast markers were in the G0/G1, G2/M, or S phases (Figure 3D), suggesting that at this early time point, the cells that entered the iN reprogramming process had exited the cell cycle and the rest of the chromatin remodeling would happen gradually without further cell division. Indeed, Tuj1^+^ cells at day 3 were EdU^−^ and did not express fibroblast markers (Figure 3E). This is consistent with a previous report,^[^ ^35^ ^]^ and implied that neuronal cell fate commitment was determined at the early stage of reprogramming.

### 2.4 ACD is Critical for iN Reprogramming

**Figure 4:**
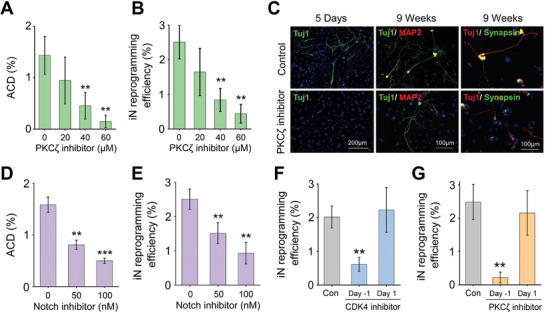
ACD is critical for iN reprogramming. A) Quantification of ACD percentage on day 1 after treatment with the PKC*ζ* inhibitor. Bar graph shows mean ± SD (*n* = 6), **p ≤ 0.01. Significance was determined by a one‐way ANOVA and Tukey's multiple comparison test. B) Reprogramming efficiency (based on the percentage of Tuj1^+^ cells on day 5 relative to the number of the cells initially seeded) in the absence and presence of the PKC*ζ* inhibitor. Bar graph shows mean ± SD (*n* = 6), **p ≤ 0.01. Significance was determined by a one‐way ANOVA and Tukey's multiple comparison test. C) Immunofluorescent images show Tuj1^+^ iN cells generated in the absence and presence of the PKC*ζ* pseudo‐substrate inhibitor on day 5 or after 9 weeks in culture and stained for mature neuronal markers, microtubule associated protein 2 (MAP2) and synapsin. Scale bar: 100 or 200 µm. D) ACD percentage on day 1 after treatment with the Notch inhibitor, DBZ. Bar graph shows mean ± SD (*n* = 6), **p ≤ 0.01, ***p ≤ 0.001. Significance was determined by a one‐way ANOVA and Tukey's multiple comparison test. E) Reprogramming efficiency in the absence and presence of the Notch inhibitor. Bar graph shows mean ± SD (*n* = 6), **p ≤ 0.01. Significance was determined by a one‐way ANOVA and Tukey's multiple comparison test. F,G) Reprogramming efficiency of BAM‐transduced fibroblasts cultured in the presence of a CDK4 inhibitor (F) or PKC*ζ* inhibitor (G) that was administered either on day ‐1 or day 1. Bar graphs show mean ± SD (*n* = 6), **p ≤ 0.01, and significance was determined by a one‐way ANOVA and Tukey's multiple comparison test.

To determine the relative contribution of ACD to iN conversion, we examined the role of atypical PKC*ζ*, a regulator of ACD, in iN reprogramming. Immunofluorescent analysis showed that PKC*ζ* was asymmetrically distributed between daughter cells, where it was highly expressed in the EdU^−^ daughter cell (Figure S6A, Supporting Information). A pseudo‐substrate inhibitor of PKC*ζ* blocked the asymmetric distribution of PKC*ζ* during cell division (Figure S6B, Supporting Information), but did not affect cell proliferation and cell viability (Figure S7, Supporting Information). In contrast, as shown in **Figure** **4**A,B, PKC*ζ* inhibition suppressed ACD and thus, iN conversion in a concentration‐dependent manner, suggesting ACD is a critical event for iN reprogramming. The maturation of iN cells was characterized by calcium fluctuation at week 6, showing similar reprogramming efficiency to the method of counting iN cells based on Tuj1 expression and cell morphology (Figure S8A,B, Movie S1, Supporting Information). PKC*ζ* inhibitor significantly inhibited the expression of mature neuronal markers after 9 weeks in culture (Figure 4C) as well as reprogramming efficiency (quantified by calcium fluctuation; Figure S8C, Supporting Information). Similarly, inhibition of Notch signaling (Figure S8, Supporting Information) that has been implicated in ACD regulation and cell fate determination,^[^ ^36^ ^]^ had little effect on cell division (with less than 100 nm of Notch inhibitor) (Figure S10A, Supporting Information), but reduced ACD and iN reprogramming efficiency in a dose‐dependent manner, suggesting a potential role of Notch signaling during iN conversion (Figure 4D,E).

To further test whether cell division at the early phase of reprogramming was important, we added cell cycle inhibitors at various time points. Adding a small molecule inhibitor of CDK4 in culture prior to Dox activation (i.e., on day ‐1 before ACD) significantly decreased the reprogramming efficiency, but adding the inhibitor on or after day 1 (i.e., after ACD peak time) had a negligible effect, suggesting that the cell division at the early phase of reprogramming was crucial for iN cell induction (Figure 4F and Figure S10B, Supporting Information). Furthermore, iN conversion efficiency was suppressed when the PKC*ζ* inhibitor was added prior to Dox activation but not after day 1 (Figure 4G). Taken together, these results further support that ACD is a deterministic step at the early phase of reprogramming.

### 2.5 Asymmetric Expression of Histone Marks during iN Conversion

**Figure 5:**
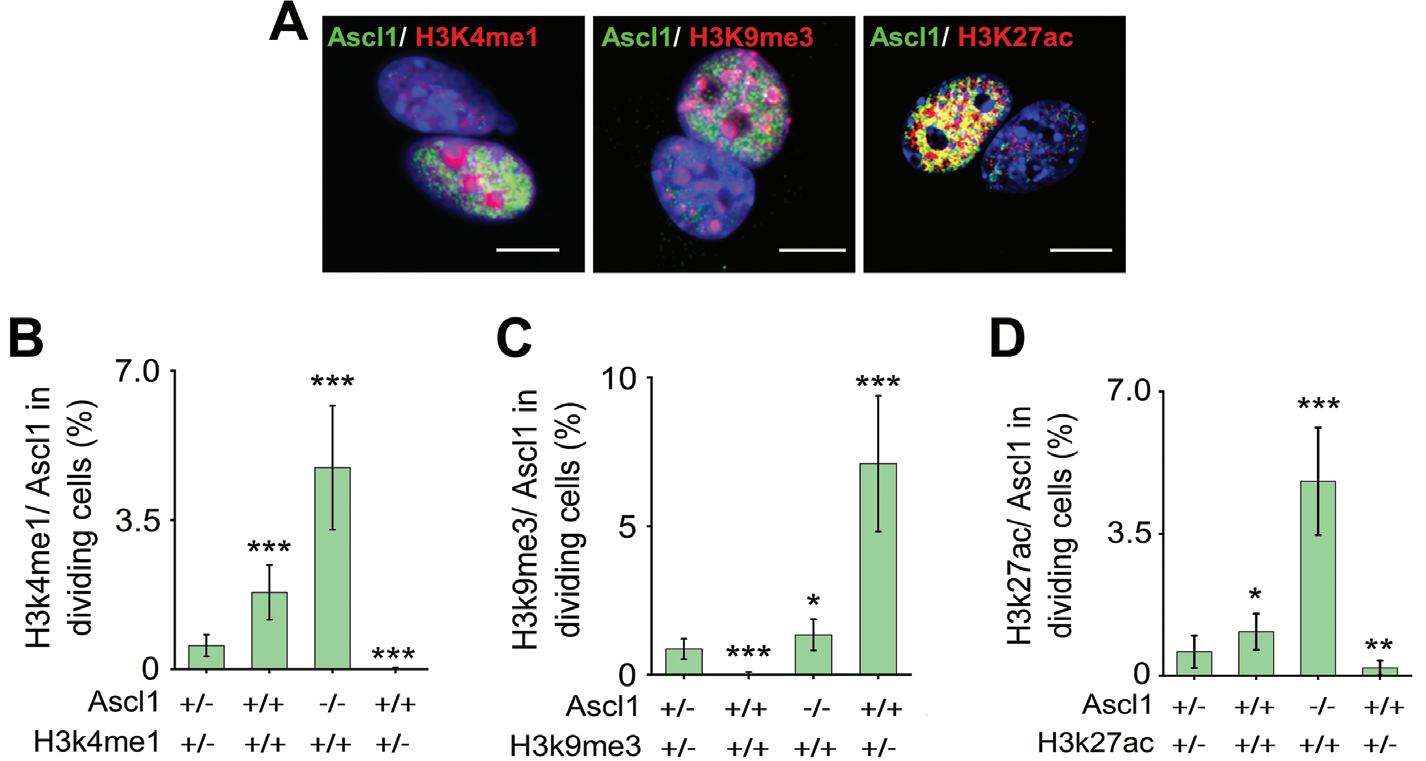
Asymmetric histone marks during iN conversion. A) Representative images of BAM‐transduced fibroblasts expressing Ascl1 and various histone marks at 24 h after Dox treatment. Scale bar: 10 µm. B–D) Quantitative analysis of the presence (+) or absence (−) of Ascl1 expression and histone marks (H3K4me1, H3K49me3, and H3K27ac) in mitotic cells on day 1. Bar graphs show mean ± SD (*n* = 6), *p ≤ 0.05, **p ≤ 0.01, ***p ≤ 0.001, and significance was determined by a one‐way ANOVA and Tukey's multiple comparison test.

To gain further insights into whether any other epigenetic asymmetry, in addition to asymmetric DNA segregation, might be occurring during ACD, reprogrammed fibroblasts were immunostained for Ascl1 and various histone marks. Interestingly, H3K4me1, H3K9me3, and H3K27ac, but not H3K4me2, H3K9ac, H3K27me3, and H3K4me3, were asymmetrically distributed between daughter cells, with higher levels of these histone marks in Ascl1^+^ daughter cells (**Figure** **5**A–D and Figure S11, Supporting Information). This finding correlates well with a previous report that a unique trivalent chromatin signature of histone modifications (i.e., H3K4me1, H3K9me3, and H3K27ac) predicts the permissiveness of Ascl1 binding to promote iN conversion,^[^ ^37^ ^]^ suggesting that the Ascl1^+^ daughter cell during ACD has an “elite” state for reprogramming.

### 2.6 ACD during iPSC Reprogramming

To determine whether ACD happened during other reprogramming processes, we examined fibroblasts transduced by Oct‐4, Sox2, KLF‐4 and c‐Myc (OSKM) viruses for iPSC reprogramming. Interestingly, we also detected ACD among the cells, and that the daughter cells inheriting the newly replicated DNA became Oct‐4^+^ (Figure S12A, Supporting Information). Furthermore, in fibroblasts with Dox‐inducible OSKM, induction of OSKM also resulted in ACD (Figure S12B, Supporting Information).

## 3 Discussion

It is widely accepted that ACD is a unique feature of stem cells and an important regulator of cell fate during stem cell differentiation. Our work, for the first time, demonstrated that ACD also happened in fibroblasts and that ACD was critical for the direct reprogramming of fibroblasts into neurons. Our results also revealed that ACD happened at the early phase (mostly day 1) and cell fate was determined after one cell division, which provides the first direct evidence of a deterministic step for cell conversion. Our single‐cell RNA sequencing analysis further demonstrated that iN precursors were in G0/G1 phase, which is in agreement with a previous finding that forced expression of Ascl1 caused cells to exit the cell cycle.^[^ ^35^ ^]^ Whether an ACD‐derived Ascl1^+^ daughter cell can develop into a mature neuron may depend on the post‐mitotic epigenetic remodeling process.

Ascl1^+^ daughter cells following ACD appear to have an “elite” state for reprogramming. In addition to the higher levels of H3K4me1, H3K9me3, and H3K27ac, both EdU pulse‐chasing and 5‐hmC staining showed that Ascl1^+^ daughter cells had newly replicated DNA strands and that the parental strands were inherited by the daughter cells with a fibroblast phenotype. Interestingly, pulse‐chasing also showed that most Tuj1^+^ cells on day 3 had newly replicated DNA strands. Since iN precursors stopped cell division following transgene expression, Ascl1^+^ daughter cells of ACD could be a major contributor to iN cells. It is possible that new DNA strands allow for easier integration and expression of transgenes to initiate ACD and that the new DNA strand may be more permissive for Ascl1 binding and chromatin remodeling during the G2/M phase to initiate iN reprogramming. What happens to the fibroblasts with forced expression of Ascl1 and symmetric division or no division? It is likely that these cells maintain most of the fibroblast epigenome, which presents a higher barrier for cell reprogramming. This possibility, together with the observation of micronuclei formation, may explain the low efficiency of iN conversion regardless of the high efficiency of transgene expression (Figure S1, Supporting Information). Therefore, we propose an “asymmetric reprogramming hypothesis”: cells with new DNA strands after ACD may have more epigenetic plasticity for reprogramming. Indeed, we have found that ACD occurred during the early phase of iPSC reprogramming (Figure S12, Supporting Information), but whether ACD happens in other reprogramming processes remains to be determined. For example, the chemical reprogramming approach and reprogramming of human cells should be investigated in future studies. Furthermore, DNA barcoding can be utilized to enable single‐cell lineage tracing and daughter cell barcoding,^[^ ^15^, ^38^, ^39^, ^40^, ^41^ ^]^ which will broaden our knowledge of the ACD mechanism during cell reprogramming.

Another unanswered question is the cause of ACD during cell reprogramming. It is possible that the cell types capable of ACD, the integration of the reprogramming factors, epigenetic changes in ACD, and many signaling pathways collectively contribute to the formation of elite cells through ACD. Interestingly, our single‐cell analysis showed that a subpopulation of iN precursors expressed Delta III, a Notch ligand, while some fibroblasts expressed Notch 2 (Figure S13, Supporting Information). It is not clear whether these changes have any effects on the reprogramming process. Further pathway analysis in cells undergoing ACD may provide more insight.

In summary, our discovery sheds light on the mechanisms of cell fate specification during cell reprogramming, which not only opens new avenues for further mechanistic studies, but also offers new opportunities to engineer cell fate and improve the efficiency of cell reprogramming.

## 4 Experimental Section

##### Fibroblasts Isolation, Culture, and Reprogramming

Fibroblasts were isolated from ear tissues of adult C57BL/6 mice (1 month old) and expanded in fibroblast medium: DMEM (Gibco, 11 965), 10% fetal bovine serum (FBS; Gibco, 26 140 079), and 1% penicillin/streptomycin (GIBCO, 15 140 122). For all experiments, passage‐2 cells were used and synchronized upon reaching 80% confluency using DMEM with 1% FBS for 24 h before the transduction with viruses containing BAM constructs. Fibroblasts were transduced and seeded onto glass slides coated with 0.1 mg mL^−1^ fibronectin (ThermoFisher, 33 016 015) overnight at a density of 2000 cells cm^−2^. The following day (i.e., day 0), the medium was replaced with MEF medium containing Dox (2 ng mL^−1^, Sigma) to initiate the expression of the transgenes and thus, reprogramming. Twenty‐four hours later (i.e., day 1), cells were cultured in N2B27 medium: DMEM/F12 (Gibco, 11 320 033), N‐2 supplement (Gibco, 17 502 048), B‐27 supplement (Gibco, 17 504 044), 1% penicillin/streptomycin, and Dox (2ng mL^−1^), and half medium changes were performed every 2 days. On day 5, cells were fixed and stained for Tuj1 to determine the reprogramming efficiency. iN cells were identified based on positive Tuj1 staining and neuronal morphology. The reprogramming efficiency was determined as the percentage of iN cells on day 5 relative to the number of the cells initially seeded. For long‐term studies where maturation and functionality of the iN cells were examined, cells were kept in culture for 9 weeks and small molecule inhibitors were only added during the first week of reprogramming.

Reprogramming of iPSC from wild‐type fibroblasts was performed as described previously.^[^ ^42^ ^]^ In addition, fibroblasts were also isolated from R26^rtTA^;Col1a1^4f2A^ mice (Jackson Laboratory, 0 1104), which expressed the Dox‐inducible polycistronic 4F2A cassette (four mouse reprogramming genes OSKM from the Col1a1 locus). To initiate iPSC reprogramming in these fibroblasts with inducible OSKM, Dox was added to the media.

##### Lentiviral Production and Transduction

Dox‐inducible lentiviral vectors for Tet‐O‐FUW‐Brn2, Tet‐O‐FUW‐Ascl1, Tet‐O‐FUW‐Myt1l, Tet‐O‐FUW‐EGFP, N‐terminal‐tagged eGFP‐Ascl1, and FUW‐rtTA plasmids were used to transduce fibroblasts for ectopic expression of Brn2, Ascl1, Myt1L, GFP, eGFP‐Ascl1, and rtTA. The STEMCCA lentiviral vector was used for the ectopic expression of OSKM.^[^ ^42^ ^]^ Lentivirus was produced by using established calcium phosphate transfection methods, and Lenti‐X Concentrator (Clontech, 631 232) was utilized to concentrate viral particles according to the manufacturer's protocol. Stable virus was aliquoted and stored at −80 °C. Fibroblasts were plated and synchronized for 24 h before viral transduction in the presence of polybrene (8µg mL^−1^; Sigma, H9268). Cells were incubated with the virus for 24 h before being seeded onto fibronectin‐coated coverslips.

##### EdU Labeling and Staining

EdU labeling and staining was performed using the Click‐iT EdU Alexa Fluor 488 Imaging Kit (ThermoFisher, C10337) according to the manufacturer's protocol. Briefly, BAM‐transduced fibroblasts were incubated with 10µm EdU for 3 h before sample collection. Samples were fixed with 4% paraformaldehyde (Electron Microscopy Sciences, 15 710) for 15 min at room temperature, washed twice with 3% bovine serum albumin (BSA; Fisher, BP1600) in phosphate‐buffered saline (PBS) and permeabilized with 0.5% Triton X‐100 (Sigma, T8787) in PBS for 20 min. Samples were then washed twice with 3% BSA in PBS followed by incubation with the EdU cocktail reaction for 30 min. After washing twice with 3% BSA in PBS, samples were stained with 4′,6‐diamino‐2‐phenylindole (DAPI; Invitrogen, D3571) for 10 min to identify nuclei. DAPI staining was also utilized to detect micronuclei formation after lentiviral transduction. The micronucleus percentage was determined as the percentage of micronuclei relative to the number of cells seeded. For pulse‐chase analysis, fibroblasts were incubated with media containing 5µm EdU for 3 days prior to BAM transduction.

##### Immunofluorescence Staining

Samples collected for immunofluorescence staining at the indicated time points were washed once with PBS and fixed in 4% paraformaldehyde for 15 min. Samples were washed three times with PBS for 5 min each and permeabilized using 0.5% Triton X‐100 for 10 min. After three subsequent PBS washes, samples were blocked with 5% normal donkey serum (NDS; Jackson Immunoresearch, 01 700 0121) in PBS for 1 h. Samples were incubated with primary antibodies (Refer to Table S1, Supporting Information) in antibody dilution buffer (1% NDS + 0.1% Triton X‐100 in PBS) for either 1 h or overnight at 4 °C followed by three PBS washes and a 1 h incubation with Alexa Fluor 488‐ and/or Alexa Fluor 546‐conjugated secondary antibodies (Molecular Probes). Nuclei were stained with DAPI in PBS for 10 min. For co‐staining of EdU with other markers, samples were first stained for EdU followed by the immunostaining procedure from the blocking step. Epifluorescence images were collected using a Zeiss Axio Observer Z1 inverted fluorescence microscope and analyzed using ImageJ.

For DNA methylation staining, samples were fixed with ice‐cold 70% ethanol for 5 min followed by three PBS washes. Samples were then treated with 1.5m HCl for 30 min and washed thrice with PBS. The immunostaining procedure proceeded from the donkey serum blocking step as aforementioned.

##### ACD and Cell Cycle Inhibition Assays

To detect the drug effects on ACD, BAM‐transduced fibroblasts were treated with the PKC*ζ* pseudo‐substrate inhibitor (Cayman chemical, 799764‐07‐1) at the indicated concentrations for 24 h prior to the addition of Dox. The inhibitor was then administered in a MEF medium containing Dox for 24 h followed by the neuronal induction medium for the remainder of the experiment. Parallel conditions with DMSO served as a control. ACD was detected via EdU staining based on a 3‐h EdU labeling, and ACD percentage was determined as the percentage of asymmetrically dividing cells after Dox treatment for 24 h (unless otherwise indicated) relative to the number of cells seeded. Similar experiments were performed using the Notch inhibitor, dibenzazepine (DBZ; Cayman chemical, 14 627).

To determine the effect of cell cycle inhibition on iN reprogramming, CDK4 inhibitor (1µm; Cayman chemical, 546102‐60‐7) was used to inhibit the cell cycle in the G0/G1 phase. BAM‐transduced fibroblasts were treated with the small molecule inhibitors (i.e., CDK4 inhibitor and PKC*ζ* pseudo‐substrate inhibitor) either on day ‐1 (i.e., in MEF medium and 12 h before the addition of Dox) or day 1 (i.e., in N2B27 medium and 24 h after the addition of Dox) and then throughout the first week of reprogramming. On day 5, samples were fixed, stained, and the reprogramming efficiency was determined.

##### Cell Viability Assays

Fibroblasts were plated and allowed to attach overnight. The following day, cells were treated with small molecule inhibitors for 12 or 24 h (as indicated) before cell viability was assayed using the PrestoBlue Cell Viability Reagent (Invitrogen, A13261) according to the manufacturer's protocol. Cells were incubated with the PrestoBlue Reagent for 2 h. Results were normalized to control (i.e., no inhibitor) samples.

##### Calcium Imaging

BAM‐transduced fibroblasts were cultured for 6 weeks before being characterized for intracellular calcium signals using Fluo‐4, AM (Invitrogen, F14201) according to the manufacturer's protocol. Briefly, Fluo‐4 AM (2µm) was diluted in phenol‐red free conditioned media, and cells were incubated with the conditioned media plus the calcium indicator for 1 h in a 37 °C humidified CO_2_ incubator. Just before imaging, cells were washed with 37 °C pre‐warmed Hank's balanced salt solution (HBSS) and incubated with 37 °C pre‐warmed phenol red‐free media. Green fluorescence intensity of Fluo4 was recorded using a fluorescence microscope (BZ 7000, Keyence, Japan) in 7.4 frames per second using a 10X objective on a 37 °C heating plate. Entire fields of view (1360‐by‐1024 pixels) were segmented as 20‐by‐20 pixels, and every frame of the movies was analyzed and plotted as a time versus fluorescence intensity graph using MATLAB (MathWorks, USA). To quantify reprogramming efficiency, cells with neuron morphology and calcium sparking were counted and normalized with the number of initially seeded cells.

##### Time‐Lapse Imaging

For live‐cell time‐lapse imaging experiments, synchronized fibroblasts were transduced with Dox‐inducible N‐terminal‐tagged eGFP‐Ascl1, Brn2, and Myt1l constructs and plated on glass bottom dishes coated with fibronectin. The following day, the media was replaced with MEF medium plus Dox and imaging experiments were performed during the first twenty‐four hours after Dox addition in a temperature‐ and CO2‐controlled chamber. Images for at least eight positions per dish/well were acquired every 4 min with a Zeiss Axio Observer Z1 inverted fluorescence microscope.

##### Single Cell Preparation and Transcriptome Profiling

For single‐cell RNA sequencing analysis, 1 × 10^5^ non‐transduced and BAM‐transduced fibroblasts were collected 36 h after the addition of Dox. Briefly, cells were trypsinized and passed through a 40µm filter to ensure a single cell suspension. Single cells were re‐suspended in the appropriate buffer and introduced into 10X Chromium for single‐cell 3’ transcriptome profiling. Briefly, single cells with a specific 10x Barcode and unique molecular identifier were generated by partitioning the cells into Gel Bead‐In‐Emulsions. Subsequent cDNA sequences with the same 10x Barcode were considered as sequences from 1 cell. The library generated by 10X Chromium machine was then sequenced on a NextSeq500 with the high output kit setting as 26 bp Read1 and 58 bp Read2. Sequencing depth was set to be 40 000 reads per cell.

##### Raw Data Processing

Raw sequencing data were converted to fastq format and demultiplexed using bcl2fastq v2 with default parameters. The resulting fastq files were then processed using CellRanger V3, using mm10 as the reference genome. The read count matrix generated by CellRanger was then analyzed using Seurat V3.

##### Data Processing

Together, 16 437 genes were detected across 11 109 single cells. Cells were further filtered by the number of genes detected (with at least 200 genes but no more than 6000 detected) and the percentage of reads mapped to mitochondrial genome out of total reads (less than 10%). 8803 cells passed these quality control. The read count data were then normalized to remove unwanted variables between cells, including those by the sequencing depth and percentage of mitochondrial reads. The normalized expression data were then used for subsequent analysis.

##### Dimensional Reduction, Clustering, Differential Gene Expression Analysis, and Single Cell Trajectory Analysis

PCA was performed using normalized expression data. Only highly variable genes (*n* = 3007) were used for PCA. To perform UMAP analysis, the first 20 PC scores for each cell were used. The clustering analysis was performed by FindNeighbors and FindClusters functions implemented in the Seurat package, with dims = 20 and resolution = 0.6. The marker genes of each cluster were identified using FindAllMarkers function with default parameters. Single‐cell trajectory analysis was performed using Monocle2 with 8803 cells and all 116 437 expressed genes. Default parameters were used for dimensional reduction, clustering, cell ordering, and trajectory visualization when performing Monocle analysis. The clusters were categorized based on the gene expression file, especially neuronal genes and fibroblasts genes. In addition, scMCA was performed for annotation as previously described.^[^ ^43^ ^]^

##### Statistical Analysis

All data were presented as mean ± one standard deviation (SD). Comparisons among values for groups greater than two were performed using a one‐way analysis of variance (ANOVA) followed by a Tukey's post‐hoc test. For two‐group analysis, a two‐tailed, unpaired Student's *t*‐test was used to analyze differences. For all cases, significance level *α* = 0.05 was set with a 95% confidence to detect a significant difference, and *p*‐values less than 0.05 were considered statistically significant. Origin 2018 software was used for all statistical evaluations. The sample size *n* was 6 (*n* = 6) in most of the experiments unless otherwise specified. For the quantitative analysis of cell proliferation, micronucleus formation and ACD during the first week of reprogramming, *n* = 4. For cell viability assay, *n* = 3, where all experimental groups were compared with (normalized by) untreated control.

## Conflict of Interest

The authors declare no conflict of interest.

## Author Contributions

Y.S. and S.L. designed the experiments. Y.S., J.S., P.W., and S.H. performed the experiments. Y.S., J.S., X.Z., S.H., and S.L. analyzed the data. Q.A. performed the single‐cell RNA sequencing data analysis. Y.S., G.F., L.L., L.Y., and S.L. contributed to data interpretation and discussion. Y.S., J.S., and S.L. wrote the manuscript.

## Supporting information

Supporting Information

Supporting Information

## Data Availability

The authors declare that all data supporting the findings of this study are available within the paper and supplementary information files.
