# Supplementary material for: Asymmetric Cell Division of Fibroblasts is An Early Deterministic Step to Generate Elite Cells during Cell Reprogramming
Source: Adv Sci (Weinh). 2021 Feb 25;8(7):2003516. doi: 10.1002/advs.202003516 (PMC8025021; doi:10.1002/advs.202003516)
Supplement: Supplementary file 1 — Supporting Information [file ADVS-8-2003516-s002.pdf]

© 2021 Wiley-VCH GmbH

ADVANCED SCIENCE
Open Access

Supporting Information

for *Adv. Sci.,* DOI: 10.1002/advs.202003516

Asymmetric Cell Division of Fibroblasts is An Early Deterministic Step to Generate Elite Cells during Cell Reprogramming

*Yang Song, Jennifer Soto, Pingping Wang, Qin An, Xuexiang Zhang, SoonGweon Hong, Luke Lee, Guoping Fan, Li Yang, and Song Li**

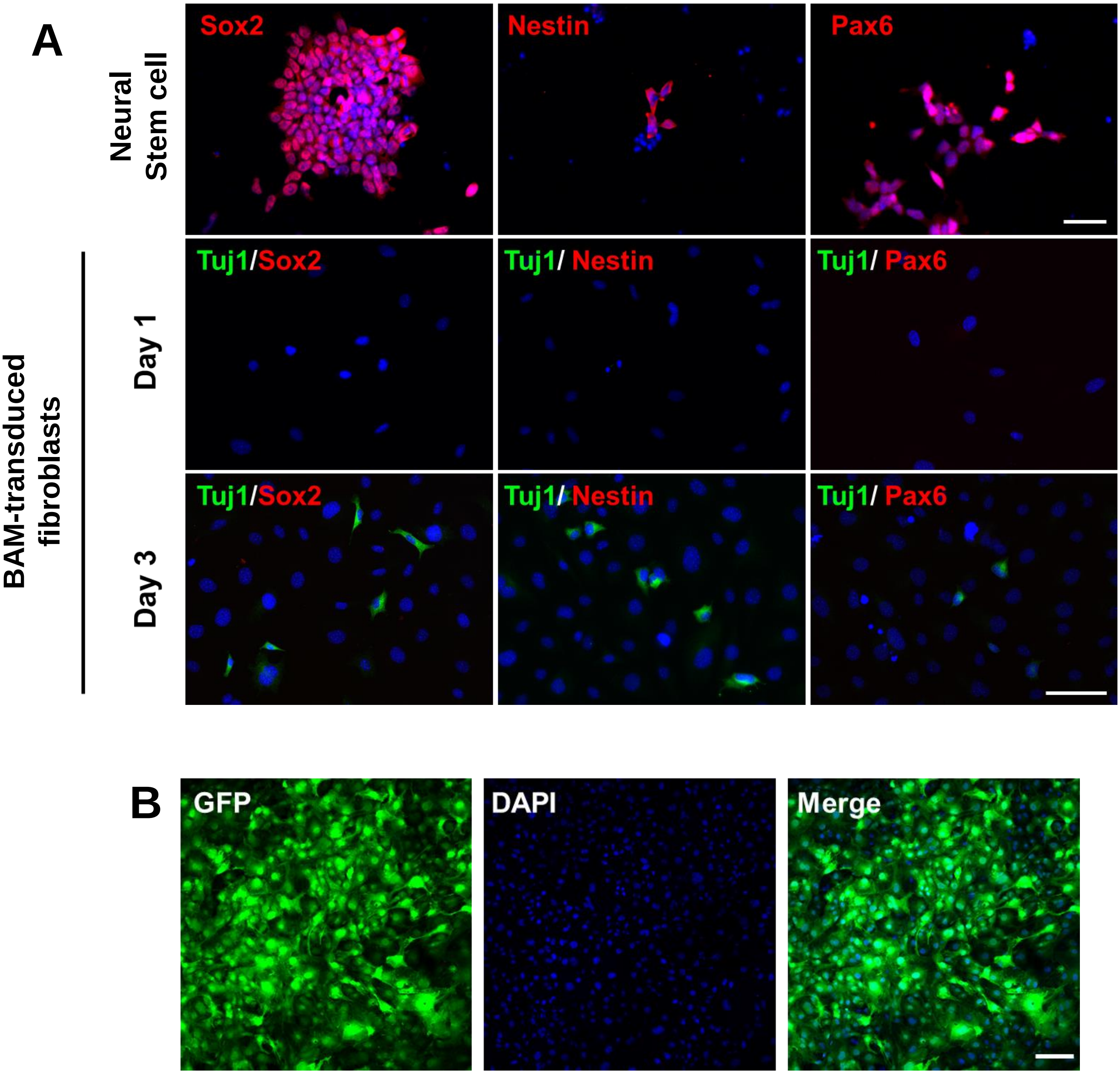

Fig. S1. Cells undergoing ACD were negative for markers of neural stem cells (NSCs). (A) NSCs derived from iPSCs (as positive control) and BAM-transduced fibroblasts were stained for NSC markers including Sox2, Nestin and Pax6 and neuronal marker Tuj1. Scale bar, 50 μm. **(B)** GFP expression in cells transduced with a GFP-lentivirus showing high efficiency. Scale bar, 100 μm.

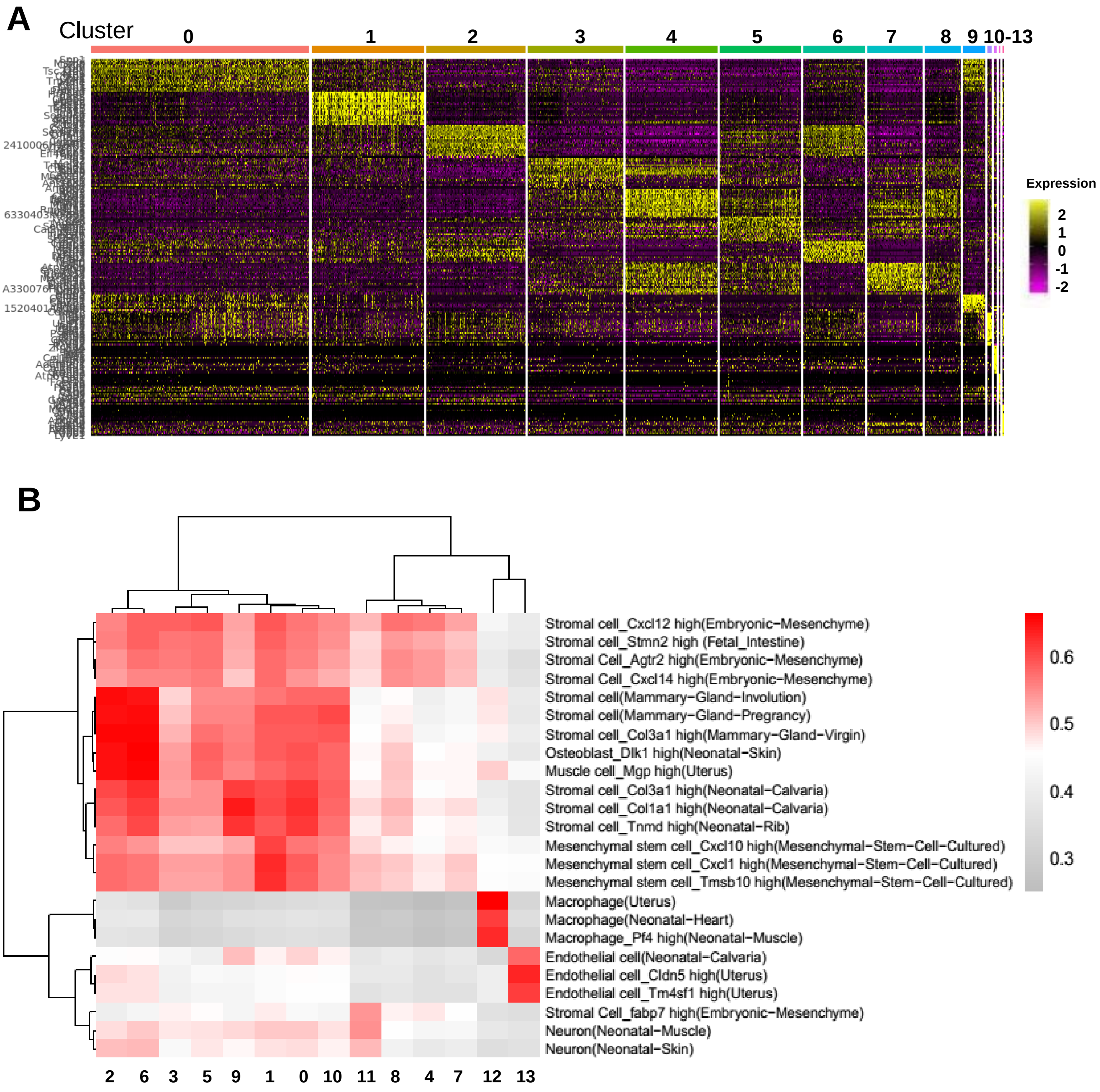

Fig. S2. Differential gene expression in cell clusters based on single cell RNA sequencing. (A) Heatmap showing expression of top10 marker genes for each cluster. **(B)** scMCA analysis of the cell clusters.

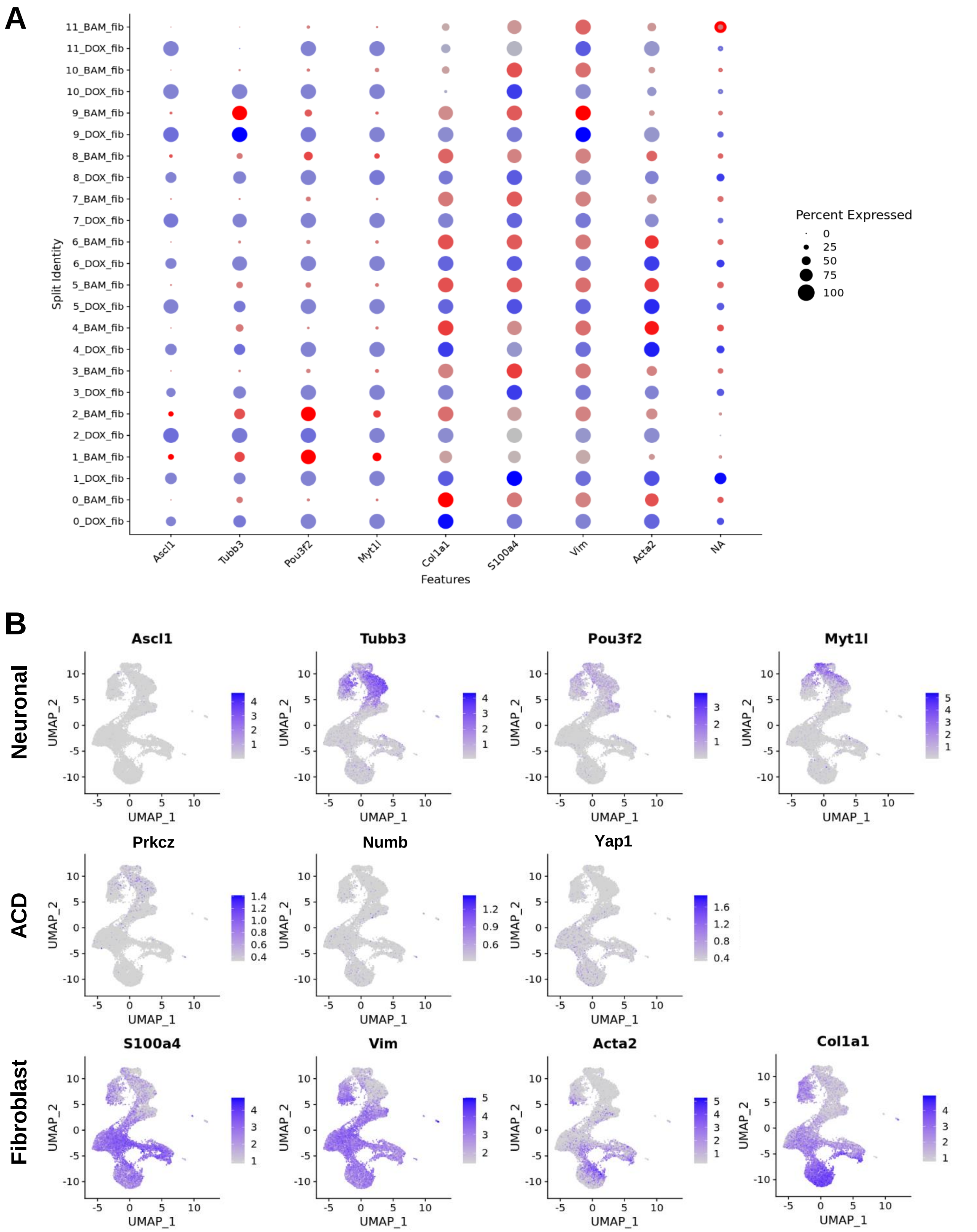

Fig. S3. Differential gene expression in cell clusters based on single cell RNA sequencing. (A) Dot plot showing the gene expression of selected markers of neurons, fibroblasts and ACD in each cell cluster. **(B)** Scatter plots showing the expression level (log10(RPM+1)) of selected genes on UMAP plots. Each dot represents a single cell. The color of dot represents the expression level of this gene in the cell.

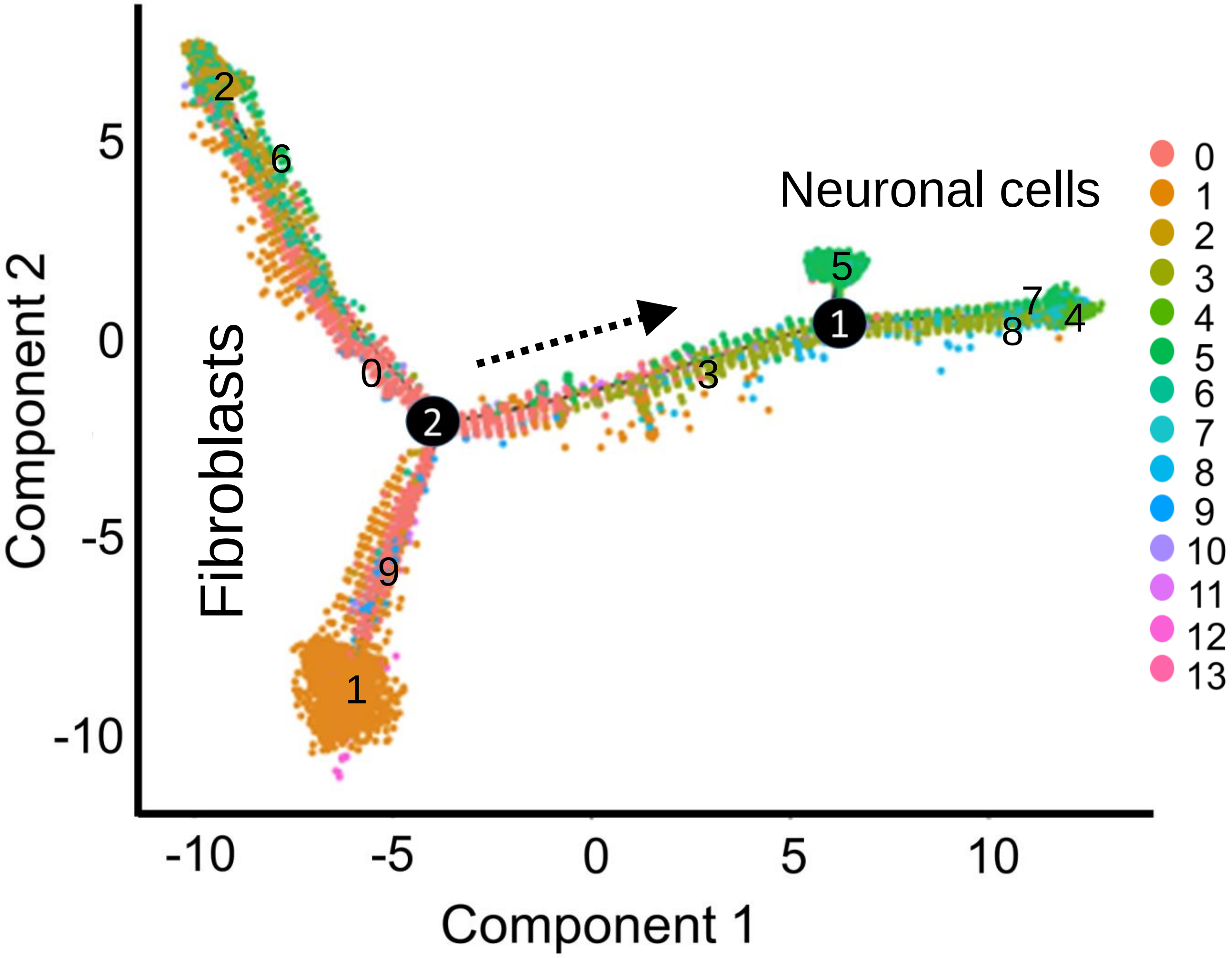

Fig. S4. Single cell trajectory constructed using Monocle2 based on all expressed genes. Monocle2 classified cells into 13 “States” for BAM-transduced fibroblasts and non-transduced fibroblasts treated with Dox. Different colors represent different states/clusters.

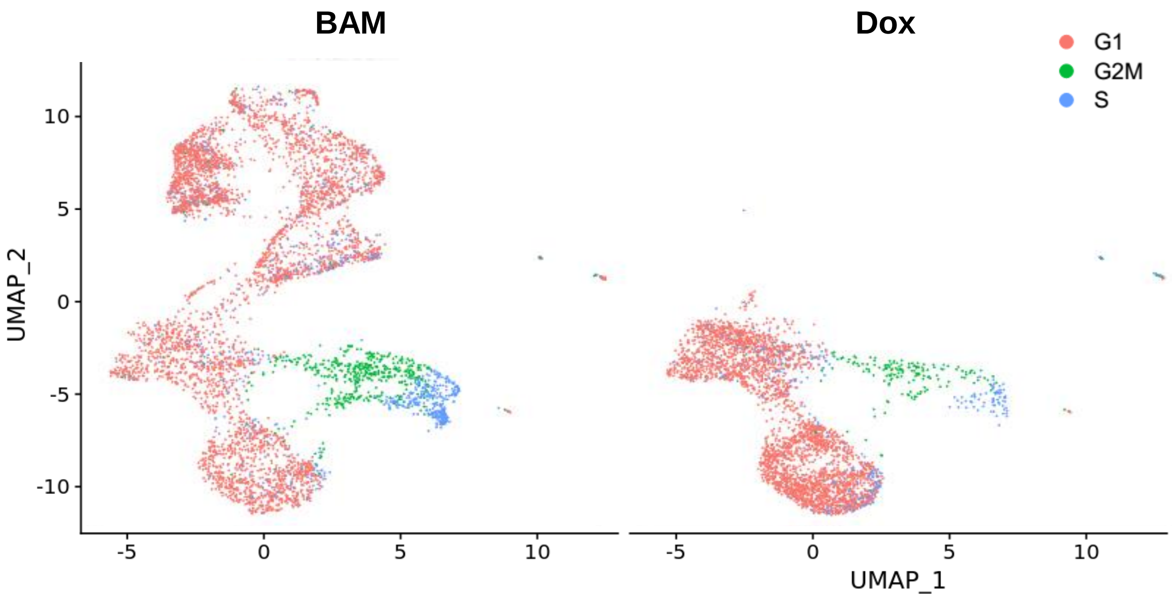

Fig. S5. Cells with a neuronal phenotype were in the G0/G1 phase whereas cells expressing fibroblast genes were present in all phases of the cell cycle. Cell-cycle phase was inferred by using CellCycleSCoring function implemented in Seurat v3 package. Visualization of cell cycle for both BAM-transduced fibroblasts (left) and non-transduced fibroblasts treated with Dox (right) on UMAP plots.

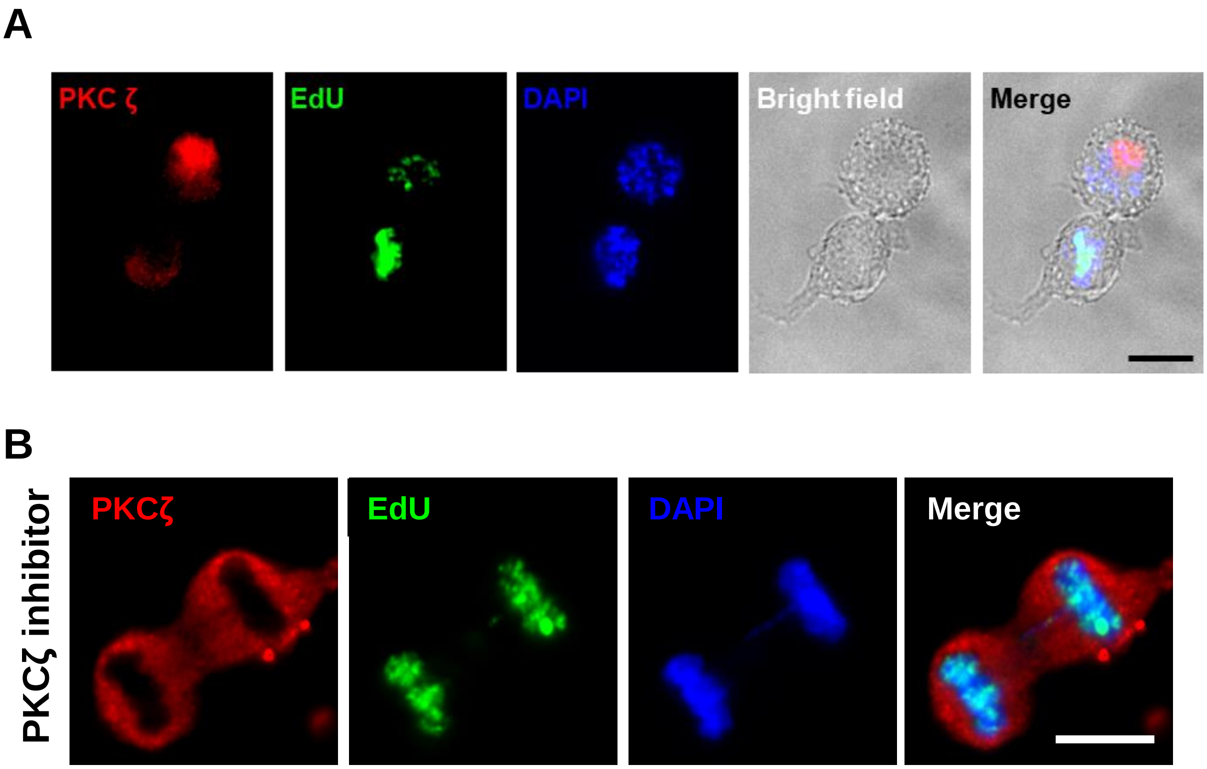

Fig. S6. PKCζ expression during ACD. (A) Immunofluorescent images show PKCζ staining and EdU labeling in dividing BAM-transduced fibroblasts at 24 hours after Dox treatment. Scale bar, 20 μm. **(B)** Immunofluorescent images show PKCζ staining and EdU labeling in dividing BAM-transduced fibroblasts treated with the PKCζ pseudosubstrate inhibitor at 24 hours after Dox treatment. Scale bar, 20 μm.

A

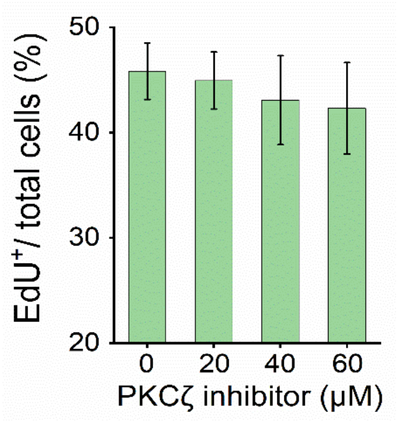

B

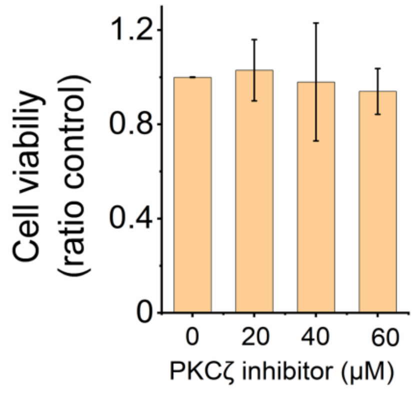

Fig. S7. Effect of PKCζ inhibition on cell proliferation and viability. (A) Quantification of cell proliferation after treatment with increasing concentrations of the PKCζ pseudo-substrate inhibitor for 24 hours (with 3-hour EdU labeling). Bar graph shows mean ± SD (n=3). Statistical significance was determined by a one-way ANOVA and Tukey's multiple comparison test. (**B)** Cell viability of fibroblasts cultured in the presence of various concentrations of the PKCζ pseudosubstrate inhibitor for 24 hours as determined by the PrestoBlue® Cell Viability Reagent. Bar graph shows mean ± SD (n=3). Statistical significance was determined by a one-way ANOVA and Tukey's multiple comparison test.

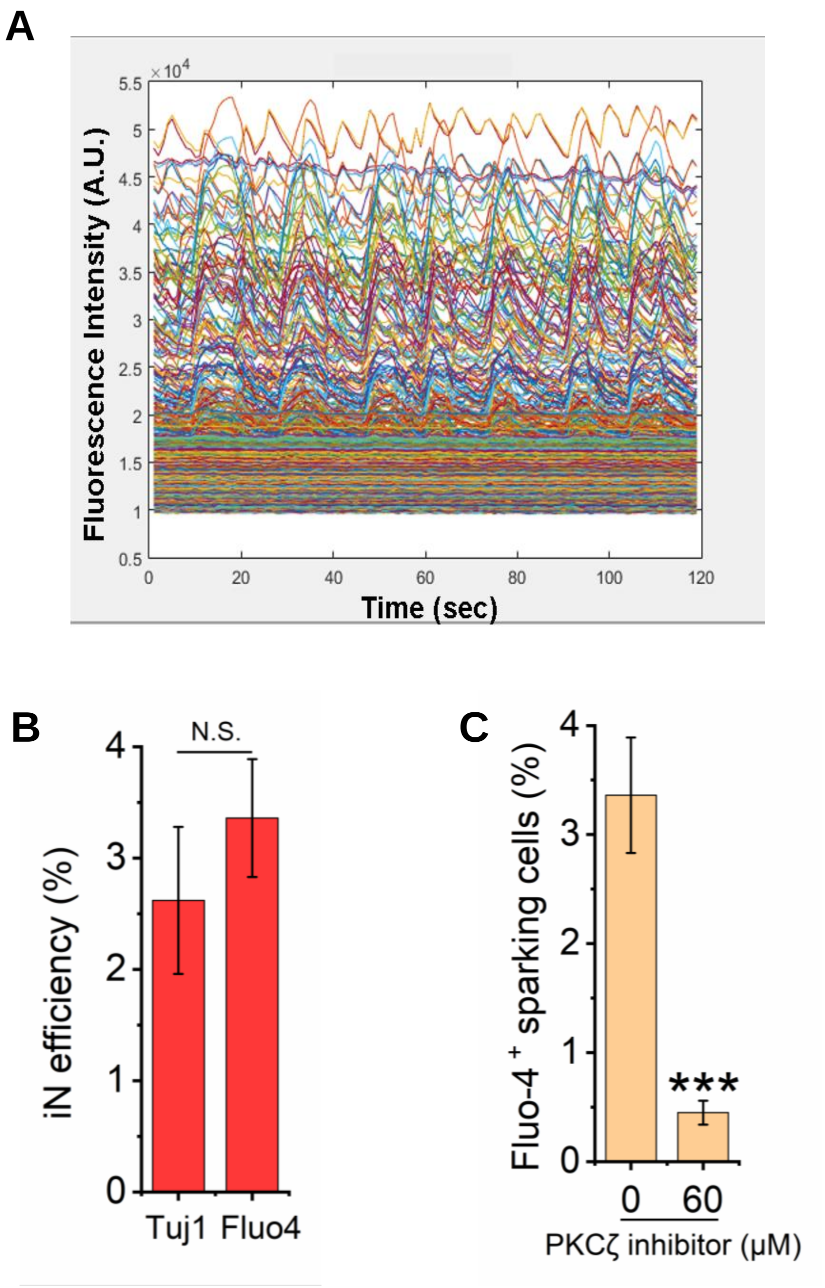

Fig. S8. Calcium fluctuation in reprogrammed cells showing the maturation of iN cells. (A) Quantification of intracellular calcium fluctuations based on the fluorescence intensity of Fluo4-AM versus time corresponding to Movie S1. iN cells displayed calcium oscillations whereas non-reprogrammed fibroblasts within the same culture did not (flat lines towards the bottom of the graph). **(B)** Quantification of the percentage of $Tuj1^+$ cells and $Fluo4^+$ cells that were producing calcium sparks from BAM-transduced fibroblasts at 1 week and 6 weeks, respectively. Bar graph shows mean ± SD (n=4). N.S.: No siginifcant difference. ***p<0.001; statistical significance was determined by a two-tailed, unpaired t-test. **(C)** Quantification of the percentage of $Fluo4^+$ cells that produced calcium sparks from BAM-transduced fibroblasts in the absence or presence of the PKCζ pseudosubstrate inhibitor (60 μM) at 6 weeks (n=4). The number of sparking cells was normalized to the number of cells seeded. Bar graph shows mean ± SD (n=4; ***p<0.001). Statistical significance was determined by a two-tailed, unpaired t-test.

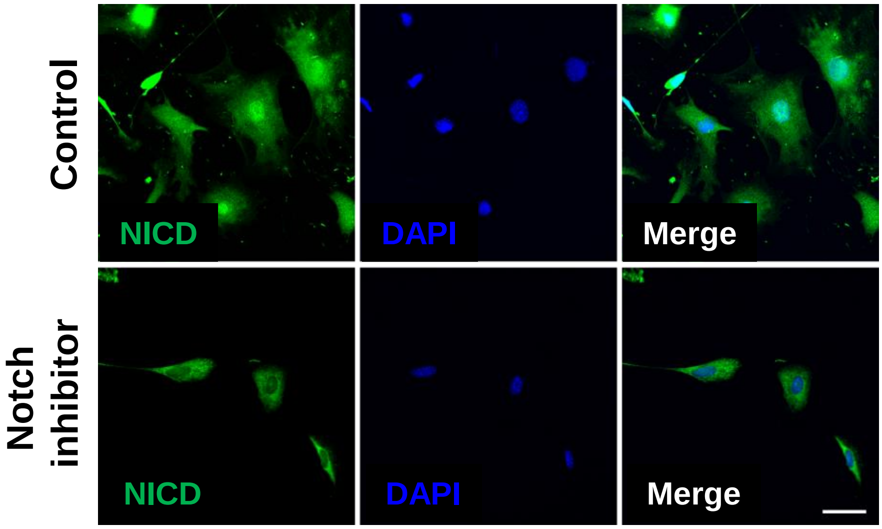

Fig. S9. Activation and inhibition of Notch pathway during iN reprogramming. Immunofluorescent images show the expression of Notch intracellular cytoplasmic domain (NICD) in BAM-transduced fibroblasts in the presence or absence (control) of the Notch inhibitor at 24 hours after Dox treatment. Scale bar, 50 µm.

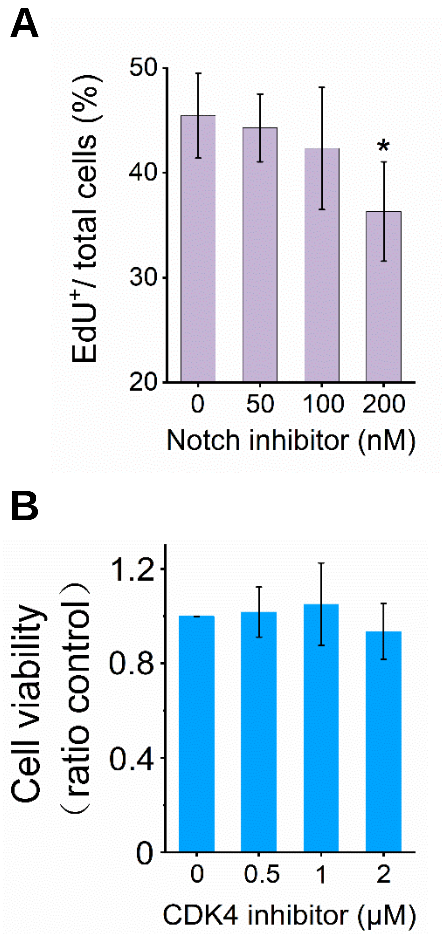

Fig. S10. Effect of Notch and cell cycle inhibition on cell proliferation and viability. (A) Quantification of cell proliferation after treatment with increasing concentrations of DBZ (Notch inhibitor) for 24 hours (with 3-hour EdU labeling). Bar graph shows mean ± SD (n=3), *$p<0.05$. Statistical significance was determined by a one-way ANOVA and Tukey's multiple comparison test. (**B)** Cell viability of fibroblasts cultured in the presence of various concentrations of a CDK4 inhibitor for 24 hours as determined by the PrestoBlue® Cell Viability Reagent. Bar graphs show mean ± SD (n=3). Statistical significance was determined by a one-way ANOVA and Tukey's multiple comparison test.

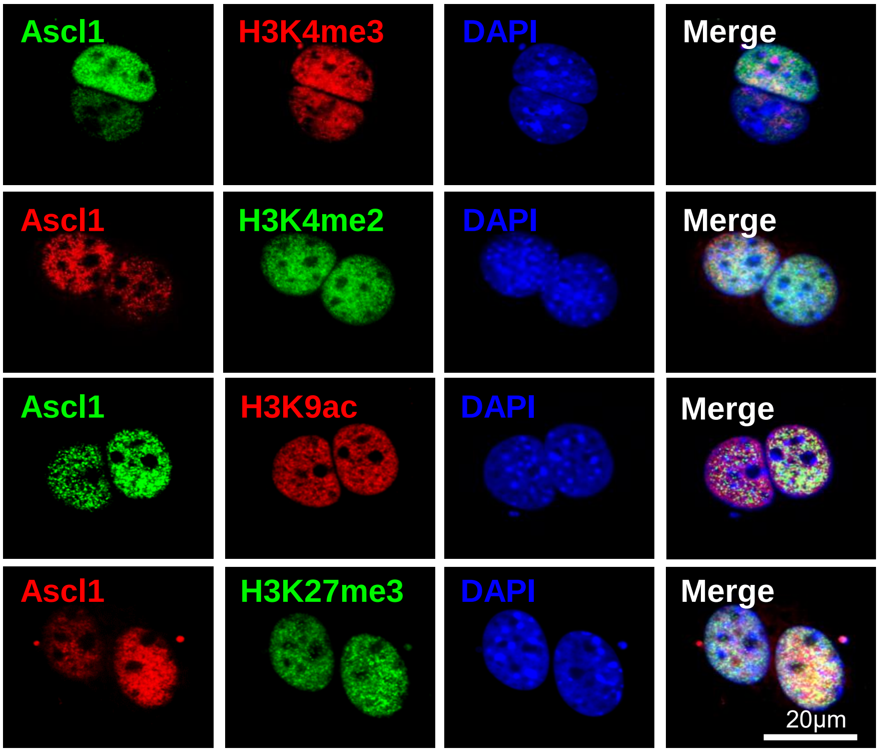

Fig. S11. Analysis of histone marks during cell division. Immunofluorescent images show the expression of Ascl1 and the level and distribution of various histone marks in dividing BAM-transduced fibroblasts at 24 hours after Dox treatment. Scale bar, 20 μm.

A

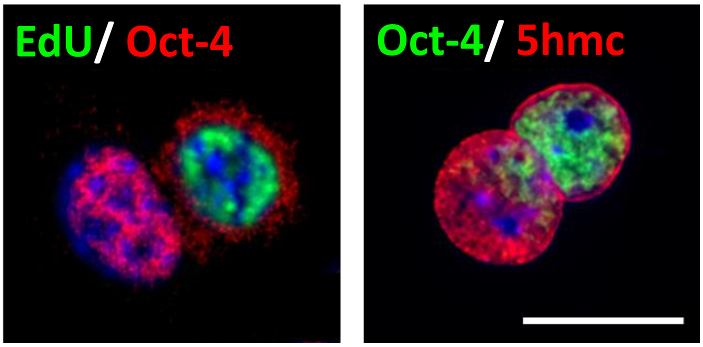

B

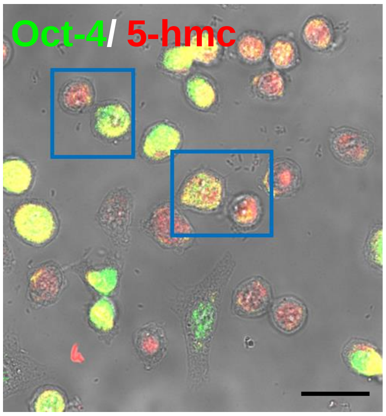

Fig. S12. ACD occurs during iPSC reprogramming. Immunofluorescent images show the expression of Oct4, EdU and 5-hmc in dividing OSKM-transduced fibroblasts at 24 hours after Dox treatment. Scale bar, 30 µm. **(B)** Immunofluorescent/phase contrast image shows the expression of Oct4 and 5-hmc in dividing fibroblast cell line with Dox-inducible OSKM at 24 hours after Dox treatment. Scale bar, 30 µm.

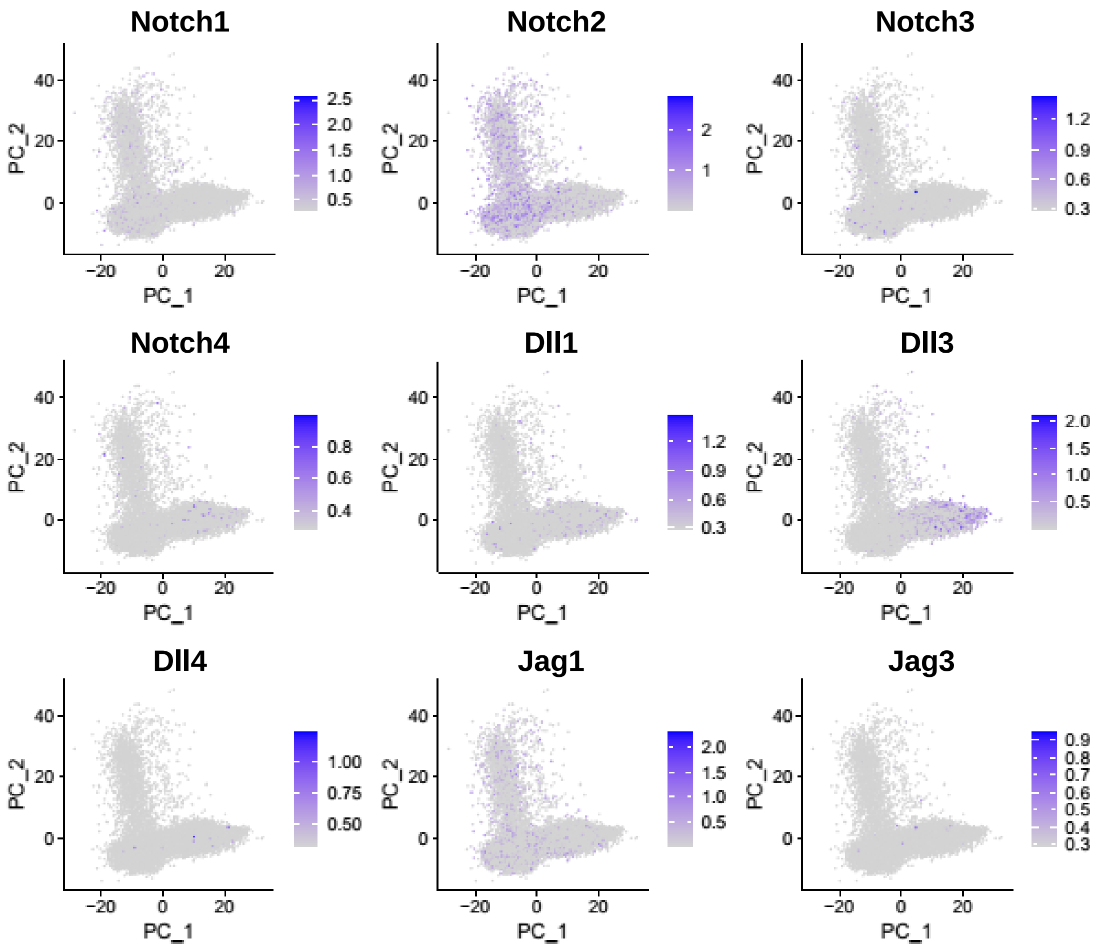

Fig. S13. ACD related pathway analyzed by single cell sequencing. PCA plots of cells expressing genes related to Notch pathway**.**

Table S1. Antibodies used in immunocytochemistry.

Antibody	Vendor	Catalog #	Dilution
NUMB	Abcam	ab4147	1:200
Ascl1	BD Biosciences	556604	1:200
YAP	Santa Cruz	sc-101199	1:100
Tuj1	Biolegend	801202	1:1000
H3K4me1	Abcam	ab32356	1:300
H3K9me3	Abcam	ab8898	1:500
H3K27ac	Abcam	ab4729	1:300
H3K4me3	Millipore	04-473	1:300
H3K4me2	Abcam	ab32356	1:200
H3K9ac	Abcam	ab4441	1:300
H3K27me3	Abcam	ab192985	1:300
5-mc	Millipore	NA81	1:300
5-hmc	ActiveMotif	39769	1:300
αSMA	Abcam	ab32575	1:300
FSP-1	Abcam	ab27957	1:200
Synapsin	Abcam	ab64581	1:100
MAP-2	Sigma	M9942	1:200
Sox2	Millipore	AB5603	1:200
Nestin	Abcam	ab27952	1:300
Pax6	Abcam	ab5790	1:300
PKCζ	Abcam	ab59364	1:300
Notch 1 (NCID)	Sigma	071232	1:300
Oct4	Santa Cruz	sc-5279	1:200

Movie Captions

Movie S1. Video of iN cells generated and labeled with the calcium indicator, Fluo-4 AM, after 6 weeks in culture.
